# Isothermal Quenching of As-Cast Medium Carbon, High-Silicon AR Steel

**DOI:** 10.3390/ma15165595

**Published:** 2022-08-15

**Authors:** Grega Klančnik, Luka Krajnc, Aleš Nagode, Jaka Burja

**Affiliations:** 1 Pro Labor d.o.o., Podvin 20, 3310 Žalec, Slovenia; 2 Department of Materials and Metallurgy, Faculty of Natural Sciences and Engineering, University of Ljubljana, Aškerčeva 12, 1000 Ljubljana, Slovenia; 3 Institute of Metals and Technology, 1000 Ljubljana, Slovenia

**Keywords:** abrasion resistant steel, as-cast, heat treatment, bainitization, bainite, martempering, martensite, steel, Thermo-Calc, JMatPro

## Abstract

Medium carbon high-silicon abrasion resistant (AR) steel was examined by performing dilatometry, light optical microscopy (LOM), scanning electron microscopy (SEM), and hardness measurements after isothermal bainitization and modified martempering and compared to direct quenching technology. A commercial thermodynamic tool was used for hardness prediction and compared to the measured one and revealed a rather good agreement for direct quenching, as was the case for isothermal holdings near to the martensite start (Ms). The predicted martensite start temperatures were in good agreement with the experimental data, the experimental value was 321 °C, while the predicted values were 324 and 296 °C. However, a higher discrepancy appeared for isothermal holding much above the martensite transition in the bainite region resulting in lower measured hardness compared to the predictions related to the actual kinetics and complexity of the formed final volume percentages of phase constituents such as bainite, martensite, and rest austenite, later as a part of unfinished bainite transformation at studied temperature. The predicted hardness values for quenching, isothermal holding at 280, 300 and 350 °C were 50.6, 50.6, 49.4 and 49.4 HRC, while the measured values were 53.3, 48.3, 48 and 43 HRC, respectively. A very good agreement between the thermodynamic prediction was achieved by comparing the measured Ms concerning prior austenite grain size as one of the crucial parameters for setting a proper heat treatment strategy of various isothermal quenchings making thermodynamic predictions for low alloyed steels a powerful tool for optimizing the heat-treating operations.

## 1. Introduction

The classical approach for the heat treatment of abrasion wear resistant (AR) steels is to use off-line (water) quenching and low temperature tempering for martensite formation and the control of the size and morphology of ɛ-carbides or alloy carbides if tempering is performed at higher temperatures, affecting the relative wear resistance of the product [1]. The relative wear resistance was also changed by using different isothermal quenching protocols, as presented for (cast) steels in ref. [2,3,4]. Quenching the products with complex geometry using water can lead to severe deformation and the presence of quench cracks. This is even more enhanced if the final product is a steel casting. The dendritic microstructure, the presence of casting defects (shrinkage porosity, intense micro-and macro-segregations, the localization of non-metallic inclusions between interdendritic arms, etc.), and coarse secondary austenite grain sizes formed after solidification, present additional crack initiation sites. The use of milder quenching media, and oil instead of water, is also limited for heavy section castings, due to the cross-sections hardness distribution. The goal of any heat treatment regardless of the chosen quench media is to produce a stable product with a reproducible outer (surface quality with minimized presence of build-up scale and decarburization depth) and inner quality (with optimal hardness distribution related to controlled microstructure transformation and consequently achieving sufficient toughness) with minimum outer residual tensile stresses and deformations.

This can be achieved by controlling the temperature uniformity during quenching with slow quenching rates, if hardenability is not an issue. More advanced approaches include introducing isothermal holding before bainite is formed, such as martempering which is (marquenching) typical for vacuum and salt quench technology of tool steels and alloyed steels or austempering, also known as bainitization for intentional bainite formation. The term bainite should be used with some precaution because the formed structure is usually without coarse carbides, and the term ausferrite is also found in the literature [5]. The mechanism of bainite formation is still a matter of scientific debate, as it is recognized as both a reconstructive [6] and a displacive phase transformation [7,8]. The complexity in the formed microstructures lies with the various growth mechanisms of ferrite from austenite, which are presented by Bhadeshia [9].

Additionally, modified martempering can be used by holding under martensite start (Ms) and much above martensite finish (Mf) [2]. Standard martempering with short isothermal holdings above Ms is carried out to achieve a uniform cross-section temperature distribution before the final quench [10]. Martempering has the benefit of having only a minor cross-section temperature difference for the final quench and also a limited self-tempering effect while achieving the maximum permitted hardness for a given carbon content compared to a direct quench technology [7].

Bainitization, on the other hand, is favored when longer heat treatment or holding times are needed, such as for heavy sections with no increased risk of ductility drop when higher amounts of carbide-free bainite are formed. Nevertheless, bainitization should be performed with a suitable chemical composition tailored for forming an ausferritic (carbide-free) microstructure. Most isothermal treatments are achieved by using salt baths. Nowadays, dry technologies are also becoming available such as dry-bainitization as DryBainTM [11] using a high-pressure gas-stream for quenching with an additional holding furnace for the bainitization of components in the production of high toughness components with high residual compressive stresses and hardness.

In the presented paper, a set of laboratory tests for bainitization and modified martempering were performed by performing dilatometric tests as a “dry-type” approach to research the isothermal heat treatment response concerning direct quench, where the target is to produce athermal martensite [12]. The findings in the presented paper are not only limited to dry-type quenching as similarities can be drawn to other quenching media. In this paper, the study was performed on a steel casting, with a typical coarse primary austenite grain (PAG) size, and a medium carbon content of 0.32 wt% C to test the possibility to obtain a uniform microstructure and sufficiently high hardness, of more than 450 HB (47 HRC) using isothermal holdings and a rather mild quenching rate for low-alloyed steel grade. Isothermal holds using salts or dry technologies are the preferred technologies because steel components are protected against heavy oxidation. Additionally, very low-temperature holdings can be considered to minimize the oxidation rate with proper care of the chosen steel composition affecting the driving force for the transformation of austenite to ferrite [13]. Isothermal bainitic treatments are usually used for high carbon steel grades and sometimes for medium and low carbon steels [14,15]. Various carbon contents and alloying elements are used for hardness and hardenability control, respectively. In principle, the maximum amount of bainite is limited, as the carbon content rises in the untransformed austenite during the bainitic ferrite (BF) formation. The carbon enriched austenite then retards the formation of BF [7,8]. Both the BF and the carbon-rich austenite volume fraction depend strongly on the chemical composition of the steels, as presented for the 0.2 and 0.3 wt% C flat steel products produced by hot rolling and on-line double-step cooling [14]. Isothermal quenching such as bainitization can be recognized as a heat treatment, where a hardness comparable to martensite can be achieved, assuming that tempering is carried out at the same temperature. However, bainitization can yield higher toughness and lower residual stresses [5] and higher elongation, determined by the tensile test [14]. There are various approaches to how to control heat treatment. In high carbon steel grades, such as 100Cr6, instead of a full austenite starting microstructure, partial martensite formation is possible (dual austenite-martensite microstructure) for the purpose to accelerate bainite transformation without affecting the bainitic kinetics [16]. It is also known that not only special heat treatment protocols (by pre-quenching) are used for accelerating the sluggish transformation into (lower) bainite, but also the addition of aluminium or cobalt [13]. Lower carbon contents are also used to influence the bainitic transformation reactions [15].

When considering the toughness of a classical quenching approach, the grain size is a major factor. The prior austenite grain size of bainitization influences the actual reaction rate and therefore the chosen holding time also importantly influences the final mechanical properties. The contradictory effect of the prior austenite grain size on the bainitic reaction rate was recognized and the difference in kinetic behavior was related to distinctions between the formed bainite microstructures [17]. The effect of austenite grain size on the bainite kinetics of isothermal bainite transformation can be expressed in a general form as defined by Matsuzaki and Bhadeshia [17]:

(1) $$X\left(t\right)=1-\exp\left(-C_{4}\times R^{m}\times t^{n}\right),$$

where X(t) represents the volume fraction of bainite Vb at the isothermal transformation time t for a given temperature T. C_4_, m, and n are constants and R is the prior austenite grain size.

The combination of good mechanical properties achieved by isothermal holdings is due to very thin bainite plates of several tens of nanometers in thickness and the fine-scale dispersion of austenite between the plates. These thin plates are responsible for very high levels of hardness [15]. Prior isothermal deformation (ausforming) is sometimes used to refine the bainitic needles and enhance the stability of the supercooled austenite [18]. It is recognized, that during bainite formation, the rest of the austenite becomes carbon enriched [6]; therefore, carbide precipitation should be limited to obtain high toughness. Silicon or aluminium (>1 wt%) are usually added to avoid carbide precipitation [8,13,17,19].

The martensite start temperature (Ms) is related to the chemical composition and also to the prior austenite grain size (PAGS), which in turn depends on the prior isothermal plastic deformation and/or austenitization temperature and holding time. This suggests that martensite transformation depends on the energy balance between the chemical driving force and the resistance exerted by the austenite against the transformation [20]. Therefore, the best way to determine the parameter Ms is experimental, as the thermodynamic predictions are usually insensitive to grain size variation and/or the chosen temperature above Ae3. The Ms obtained by thermodynamic software with no relation to austenite grain size (PAGS) should be taken as the fundamental martensite start for an infinitely large grain size. The concept of fundamental martensite start [15] can be expressed as:

(2) $$M_{S}^{0}-T=\frac{1}{b}\ln\left[\frac{1}{{\mathrm{aV}}_{\gamma }}\left\{\exp\left(-\frac{\ln\left(1-f\right)}{m}\right)-1\right\}+1\right],$$

where $M_{S}^{0}$ represents the highest (fundamental) temperature at which martensite is formed and $M_{S}^{0}-T$ is the temperature difference due to the change in austenite grain size, described as the average austenite grain volume ($V_{\gamma }$). The constants a and b are material fitting parameters and f is the first detectable volume fraction of martensite (for example 0.01). The constant m is taken as 0.05 [15].

Calculations for the formation of athermal martensite fraction (f) during quenching are described with a sigmoidal curve [18,21,22] or parabolic curve as in the case of the most common empirical Koistinen–Marburger (K–M) equation [22]:

(3) $$f=1-\exp\left(-\alpha_{m}\left(T_{\mathrm{KM}}-T\right)\right),$$

where the martensite start is described as the theoretical martensite start temperature T_KM_, or simply Ms, and T is the isothermal holding (or quench) temperature below Ms. The coefficient α_m_ is a rate parameter. An example of a martensite kinetics study using the K–M equation is found in [23]. The nature of the martensite fraction evolution also defines the volume fraction of martensite before isothermal holding begins under Ms important for the pre-quench step.

The purpose of this study was to evaluate pre-defined bainitization and modified martempering in a laboratory setting instead of a classic direct quenching approach to satisfy the minimum criteria for abrasion-resistant application, achieving high hardness with a minimum of 450 HB and limiting, at the same time, the internal quench stresses by including temperature equalization during quenching. Two commercial thermodynamic tools were used with experimentally obtained data (Thermo-Calc, Thermo-Calc Software AB, Stockholm, Sweden, and JMatPro, Sente Software, Guildford, UK)). Since the material under investigation is an as-cast steel, the heat treatment is an even more important step for achieving properties, because the as-cast material has limited options for grain refinement and is very susceptible to cracking during fast cooling.

Hardness prediction for non-continuous cooling patterns (being between CCT and TTT diagrams) can be highly demanding as already indicated by the description above. By knowing that Ms, Bs is related not only by chemical composition, but also the degree of the achieved mechanical stability of the austenite (related also to the dislocation density), achieved prior austenite grain size (presented in the results and discussion), chosen isothermal holding times and temperatures, related incomplete reactions and many more factors, including the kinetics of a given carbon content for BF (and most importantly on the thickness of BF plates, possibly changing during holding time), martensite and carbide formation has an impact on the overall volume fraction and interface density of the final microstructure and therefore on hardness. The variety of mechanism for solid phase transformation control should be taken into consideration for achieving the target steel properties and can be found in extensive reviews [24,25,26,27].

The laboratory trials were performed as sufficient experimental data are needed, such as hardness (as indication of extent of certain phase transformation, change in BF plate sizes, etc.) and microstructure interpretation which represent basic data for the majority of (as-cast) steels, so as to compare the validity of current and available models incorporated in commercial thermodynamic tools as the next generation of industrial tools for new and modern steel grade development.

## 2. Materials and Methods

The material analyzed in this paper was induction melted using argon protection and gravitationally cast into the dried and preheated sand mould to a final shape of approx. 300 × 50 × 50 mm^3^ and heat-treated without any plastic deformation. The material under investigation is a medium carbon low alloyed steel with 0.32 wt% C, similar to compositions used for direct quenched hot-rolled ultra-high-strength steel (UHSS) products and high strength carbide free bainitic steels [19]. The laboratory manufactured grade, using an induction open furnace, has an increased silicon and low aluminium content (<100 ppm Al) to minimize the presence of aluminate non-metallic inclusions. The high silicon content is usually used with approx. 2 wt% to prevent the very coarse precipitation of brittle cementite carbides [14]. Silicon has a significant effect on carbon partitioning in austenite and hinders the decomposition of the retained austenite caused by tempering and cementite formation [28]. The chemical composition is provided in Table 1. The steel melt was zirconium treated with 200 ppm Zr. Zirconium is added when the degree of desulphurization is limited and, in cases where sulphide shape control is required such as in forgings or hot-rolled products [29]. Due to low sulphur contents, such as in this case, it was primarily used for grain size control [30,31] and control over excessive shrinkage porosity for the repeatable hardness measurement presented in the paper. Zirconium is a strong nitride-forming element and can easily form nitrides, as is exemplified in this paper. For dilatometric tests and for chemical composition analysis, the samples were extracted by performing cold saw cutting at the position of 1/4T, where T is the thickness of the produced casting. The chemical composition was determined using optical emission spectrometry (OES) Agilent 5800 VDV (Agilent, Santa Clara, CA, USA) and LECO (Leco corporation, St. Joseph, MI, USA) for sulphur and carbon control.

Characteristic temperatures and transformation kinetics were studied using a quenching and deformation dilatometer DIL805A/D (TA, New Castle, DE, USA). Samples were induction heated under vacuum to austenitization temperature and cooled using pressurized argon (99.999 wt%). Heating from room temperature to 600 °C was performed at a rate of 10 °C/s and 1 °C/s from 600 °C to the austenitization temperature (920 °C). Direct quenching and quenching till holding time for bainitization or modified martempering were performed at a rate of 10 °C/s. The isothermal holding temperatures and times were 280 °C—1 h, 300 °C—2 h, and 350 °C—2 h. The holding times were chosen as upper time limits for practical industrial production (short times), where holding times are related also to the thickest section of the AR product and less to complete bainitic transformation. Afterwards, the cooling was set at 30 °C/s. It is recognized that for some steel grades, for example those forming super bainitic structures by transforming the austenite to bainite can take from 6 h up to 3 days [30]. The recorded temperature–time curves for bainite transformation observation were constructed using type S thermocouples (Pt-PtRh). Afterward, the hardness measurements were conducted using an Instron B2000 Rockwell test machine and hardness conversion tables (ISO 18265, 2013) were used. The average values are represented by using at least three individual hardness measurements.

The parabolic martensite fraction in relation to undercooling was calculated using thermodynamic commercial software JMatPro 6.1 using the Properties quenching module and General steel database and the sigmoidal curve was calculated using Thermo-Calc 2020a with a TCFE9 database. For solid-state transformation in steel, MUCG83 was also used.

Samples were metallographically prepared by performing grinding with SiC papers, then polishing with 3 to 1 μm of diamond suspension, and finally etching with 5 vol.% Nital for LOM Microphot FXA, Nikon (Nikon, Minato City, Japan), with a 3CCD video camera, Hitachi HV-C20A (Hitachi, Ltd., Tokyo, Japan) and scanning electron microscopy (ThermoFisher Scientific Quattro S field-emission SEM, ThermoFisher Scientific, Waltham, MA, USA). Polished samples were also used for hardness measurements (Instron B2000 Rockwell test machine, Instron, Norwood, MA, USA).

**Figure 1:**
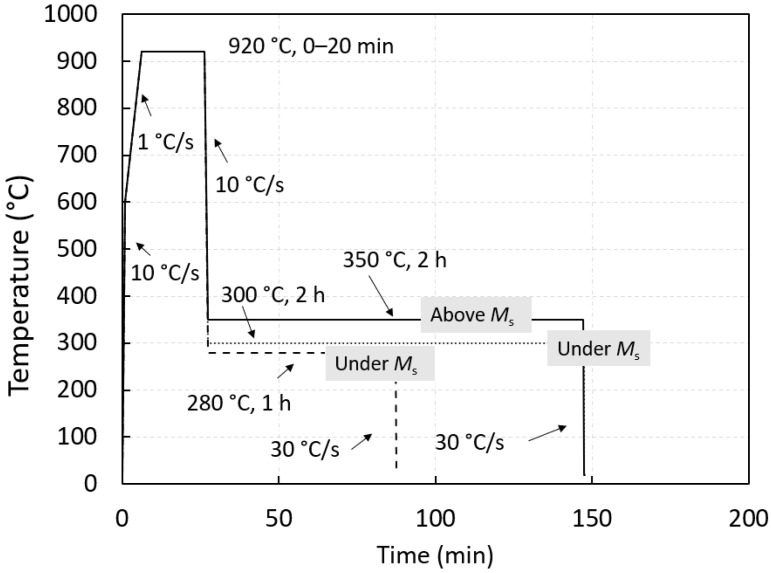
Dilatometric heat-treatment cycle. Ms is fixed to experimentally determined temperature.

The characteristic temperatures needed for laboratory isothermal heat treatment are presented in Table 2. Transformation kinetics were studied using three isotherms at 280, 300, and 350 °C with holding times of 2 h for the two highest temperatures and 1 h for the lowest temperature of 280 °C. The highest temperature is also interesting due to the current limitation of the salt bath practice [32]. For a specific set of tests, a temperature variation (900 ± 25 °C) with fixed holding austenitization times was tested for the possible refinement of the final prior austenite grain size. With a similar response in hardness and grain size for direct quench, a fixed temperature of 920 °C was chosen for isothermal quenching. Nevertheless, the grain size can influence the bainitic kinetic behaviour [17]. The heat treatment protocol is presented in Figure 1. Variation of the holding time at the austenitic temperature was set to 20 min due to the already coarse PAGS in the as-cast state.

## 3. Results and Discussion

The carbon content defines, among other alloying elements, the martensite start, Ms, and maximum expected hardness of athermal martensite. Usually, higher carbon-high silicon alloys are used for austempering or bainitization to be performed at the lowest possible temperature. Nevertheless, the negative element of such treatments is the long treatment times; therefore, a decrease in the carbon content (<0.2 wt%) has been known to increase the driving force and thus accelerate the bainitic transformation rate. This, however, also results in coarser bainite laths, but still, the low carbon steels are a promising choice for ausforming [18]. In the present paper, medium carbon steel was set with a composition of other alloying elements presented in Table 1 to prevent any high-temperature products, ferrite or perlite, during cooling to isothermal holding temperature.

### 3.1. Thermodynamic Prediction

Equilibrium characteristic temperatures Ae1 and Ae3 were determined using both commercial software Thermo-Calc (Thermo-Calc 2020a, Thermo-Calc Software AB, Stockholm, Sweden) and JMatPro (JMatPro 6.1., JMatPro*^®^*, Guildford, UK). Reasonable agreement was obtained by comparing both programs and related databases for equilibrium characteristic temperatures, Table 2. For Ae1 the value determined by JMatPro and Thermo-Calc was taken from the equilibrium phase calculation for a lower Ae1 temperature. By predicting the CCT diagram, the upper Ae1 temperature was predicted, with all carbides being soluble in the austenitic matrix in a two-phase region (γ + α) with A1 = 764 °C. The latter fits very well with the experimentally determined Ac1 = 760 °C. Overall, the experimentally determined characteristic temperatures are slightly higher than predicted related to the heating rate used for the dilatometric test. The bainite-start temperature (BS) was predicted using JMatPro with the General steel database; additionally, MUCG83 [15,33] was used for nucleation of the limited bainite start prediction. According to Table 2, the temperature interval for bainite formation was expected to be between the rather high predicted Bs = 488 and measured Ms = 321 °C. The rather high predicted Bs was a result of the low carbon content of the investigated as-cast alloy.

**Figure 2:**
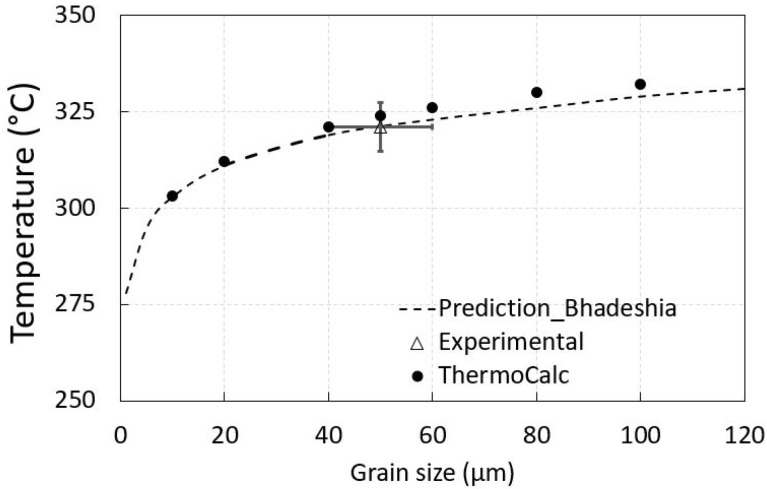
Variation of martensite start (Ms) in dependence to grain size. The prediction was done by using Equation (2).

A property model in Thermo-Calc was also used for Ms determination concerning grain size with corrected parent phase Gibbs energy addition (100 J/mol). Because the investigated sample was cast, the grain size was expected to be rather coarse compared to products that are refined by plastic deformation, such as forgings, etc. The relation between grain size and Ms is presented in Figure 2. There was a dependence of average PAGS on Ms as in the work of Yang and Bhadeshia [34] or Celada-Casero [20]. It is obvious that the isotherm holdings at 300 and 280 °C were under the experimental Ms regardless of the typical and expected prior austenite grain size distribution in the casting (from 10 to 100 µm).

**Figure 3:**
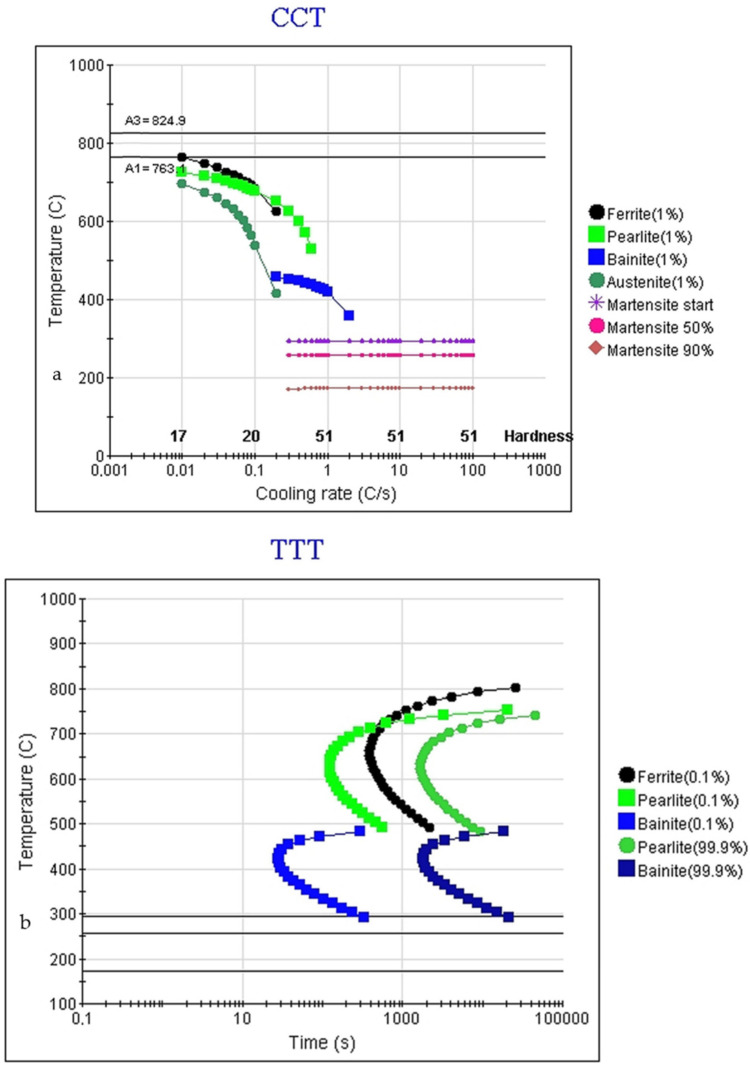
Predicted (**a**) Continuous cooling transformation diagram (using Advance CCT, JMat Pro) and (**b**) Transformation-time-temperature diagram.

**Figure 4:**
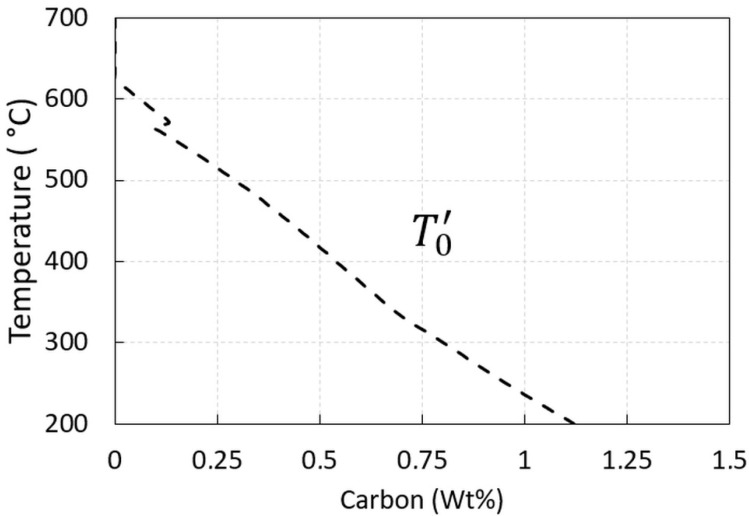
Calculated $T_{0}^{\prime }$ using MUCG 83 concerning carbon in austenite [33].

A very good agreement was also obtained for Ms with the corrected value for parent phase Gibbs energy addition determined by Thermo-Calc with the experimental one and Mucg83. The JMatPro results show the lowest value of Ms, as presented in Figure 3. Experimentally determined Ms was 321 °C. When bainitization was considered, the different carbon content in residual austenite was achieved (Figure 4) and therefore the change of Ms was expected.

**Figure 5:**
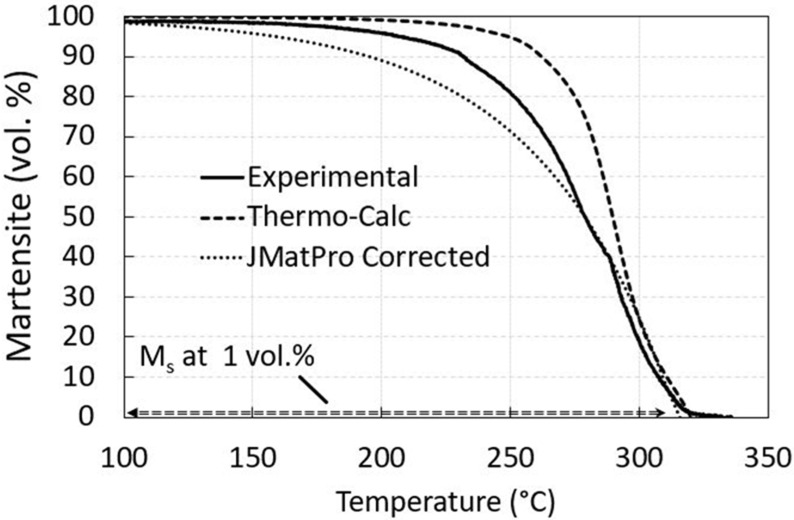
The parabolic martensitic volume fraction development concerning undercooling before isotherms by using JMatPro (Quench properties) and sigmoidal development of martensitic fraction using Thermo-Calc 2020a (TCFE9) and property model calculator based on [22] compared to experimental work.

Due to the relatively low carbon content, the martensite finish temperature (Mf) was determined to be above room temperature for all predictions and much lower than the lowest holding temperature used in the present study, especially for JMatPro calculation. Mf was also used to estimate the martensitic fraction development relative to undercooling, Figure 5. A reasonable agreement was obtained with both thermodynamic softwares regarding the experimentally determined one. For easier comparison, the martensite fraction evolution calculated by JMatPro was artificially corrected to the experimentally determined Ms value, also presented in the paper.

The Continuous Cooling Transformation (CCT) diagram calculated for as-cast AR steel using JMatPro is presented in Figure 3a. The lower bainite field crossing under continuous cooling was calculated at 2 °C/s. To avoid the bainite nose under continuous cooling conditions, a 10 °C/s cooling rate was set as is typical for the quenching of heavy sections using dry-bainitization technology. As discussed already in ref. [14], a shift is expected for the bainite region to higher temperatures and shorter times compared to the high carbon steel grades. An additional shift to shorter times is expected when the TTT diagram is considered for the absolute isothermal holdings, presented in Figure 3b. The bainite formation is expected to be under the $T_{0}^{\prime }$ line, Figure 4, and when using nominal carbon composition in the austenite this is expected to be under 488 °C and 486 °C according to MUCG83 and JMatPro, respectively (see, Table 2). It should be emphasized that in the case of the as-cast state due to micro-segregations certain variations of $T_{0}^{\prime }$ were expected and with that related *V*_*b*_ at a given isothermal holding. The maximum hardness is expected for martensite formation but in the case of JMatPro prediction, it is obvious that by continuously crossing the lower bainite region, for example at 1 °C/s, the hardness is barely changed compared to full martensite transformation (Figure 3 a) and therefore minor changes in hardness are expected also for isothermal holdings in the bainite region very near Ms.

At 350, 300, and 280 °C (Figure 4) the maximum permitted carbon levels in austenite were estimated to be 0.66, 0.81, and 0.86 wt% C, respectively. Using this value, the estimated volume fraction of bainitic ferrite was calculated (based on Equation (1) in ref. [8] to be 0.54, 0.62, and 0.65 for the carbon values in austenite, respectively. The calculated fraction of retained austenite and/or transformed austenite to martensite at room temperature is, therefore, 0.46, 0.38, 0.35, respectively. This provides information on possible blocky residual austenite formation (especially at higher isotherms), due to the increased residual austenite content before the final quench. Additionally, by using the quench module (JMatPro) at 350, 300 and 280 °C the estimated volume fraction of bainite was found to be higher with 1.00, 0.94, and 0.0005 (as being under Ms and majority at room temperature is martensite). It seems that discrepancies appear at higher holding temperatures above Ms due to the practically completed bainite transformation in the bainite region based on JMatPro prediction.

That the phase transformation is complex at given isothermal temperatures is also obvious according to the determined starting martensite fraction derived from experiments in Figure 5 with approx. 0, 20, and 49 vol.% of martensite before the isothermal bainite transformation began at 350, 300, and 280 °C. Using Thermo-Calc, the martensite fraction at a similar Ms as experimentally determined increased with undercooling as in the case of JMatPro. Due to low predicted Ms in the case of JMatPro the curve is corrected to experimental Ms, Figure 5. The satisfactory martensite fraction correlation was achieved with JMatPro and especially with Thermo-Calc with up to 40 vol.% of martensite where the martensite development was sigmoidal at later stages. However, the experimentally determined volume martensite fraction was in between both predictions.

### 3.2. Dilatometric Measurement

**Figure 6:**
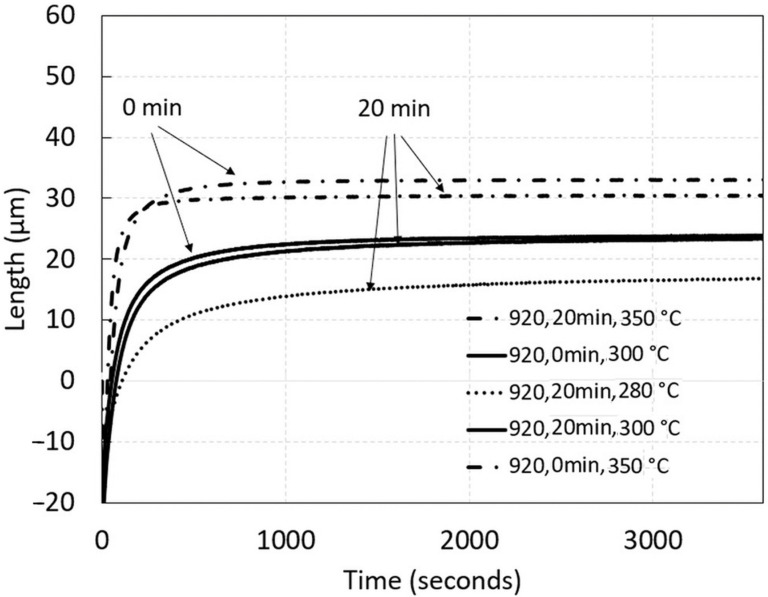
A detail of isothermal transformation kinetics with time shifts not including the incubation time for bainite start.

The transformation kinetics at isothermal holdings were observed by comparing the following three isotherms: 350, 300, and 280 °C. Interestingly, there was no measurable difference between the variation of the austenitization holding times from 0 to 20 min and the measured length change at the same holding temperature. This can be attributed to the fact that a limited change in grain size is expected between both holding times. The austenitization at 920 °C is well above the measured Ac3 with 830 °C and certain grain growth was already achieved due to the as-cast state regardless of the holding time. The grain size is expected to be changed by increasing the austenitizing process to higher temperatures or by introducing longer austenitizing times, with more than the 20 min used in this study. The continuous transformation is related to the length change of the sample, due to bainite formation. According to Figure 6, the transformation kinetics at the isothermal holdings were observed for all temperatures meaning that by using modified martempering (under Ms) the rest of austenite also transformed into (lower) bainite.

For the estimation of the isothermal bainitization progress, a clear stabilization of length change at 350, 300, and 280 °C was achieved after 25, 42, and 58 min, respectively. Nevertheless, this should be taken as an estimation only, and should be interpreted as the visual termination of isothermal transformation. The effective termination can be extended to much longer times, especially for lower temperatures, and is related to the retained austenite stabilization. However, it is seen that bainitic kinetics become enhanced at higher temperatures with a similar short incubation time, not shown here, with bainite start within 60 s for the temperature interval of interest. Based on the TTT diagram in Figure 3b the incubation time should be under 6 min for bainitization to begin. The diagram of isothermal transformation kinetics in Figure 6 is presented to distinguish all the measured curves.

### 3.3. Hardness Measurement

The hardness of the samples relates to several factors. It is known that isothermally formed retained austenite can contribute to the final hardness, for example, by transforming unstable (low carbon) austenite to martensite after cooling to room temperature, or by mechanically (strain- or stress) induced martensite transformation, the achieved actual volume fraction of austenite and relative austenite stability. The so-called “blocky” austenite forms hard phases, not only untempered martensite but also in some cases carbides, which later can create voids leading to a decrease in toughness [7,14]. Therefore, the hardness of a sample depends on the hardness and volume of the stable retained austenite, bainite (bainitic ferrite), and martensite. The latter is influenced by the carbon content in the austenite before martensitic transformation. From this point of view, a mixed microstructure (bainitic ferrite + austenite + martensite) can be expected in all the investigated samples after isothermal holds from 280–350 °C, according to the results of the transformation of kinetic progress, Figure 6. This complexity is, therefore, not easily predicted. It is also known that the “law of mixtures” is not strictly followed by the equation for carbide-free mixtures [7]:

(4) $$H=V_{\gamma }H_{\gamma }+V_{b}H_{b}+V_{M}H_{M},$$

where nominal hardness *H* is calculated by the separate hardness contributions of *H_γ_*, *H_b_*, *H_M_*, related to retained austenite, bainitic ferrite, and martensite formed after cooling and the related volume fraction *V*.

The fraction of retained austenite is related to blocky parts and also to the desired stable (high carbon) austenite films between bainitic ferrite and is directly related to the chosen isothermal holding temperature, as can be seen in Figure 4, where the extent of maximum isothermal bainitization is related and limited to $T_{0}^{\prime }$, which is the temperature where austenite and ferrite of the same composition have the same Gibbs free energies [15]. It is expected that lower amounts of unstable “blocky” austenite are formed at low-temperature isotherms, and also higher bainitic transformation volumes (*V*_*b*_) are achieved using proper holding times and, therefore, higher hardness values are achieved at lower temperatures. If sufficient unstable residual austenite exists due to limited (sluggish) bainitic kinetics with residual austenite transforming to martensite during the final quenching, the hardness trend is opposite to the statement above as the highest hardness is achieved at the maximum holding temperature with a prevailing martensite fraction [15]. It was also observed that at lower holding temperatures and the same holding times, followed by quenching, more martensite (as a part of unstable residual austenite) is formed than at higher isothermal temperatures as the rate of bainitic transformation is faster at higher temperatures and the fraction of retained austenite to form martensite is smaller [19].

**Figure 7:**
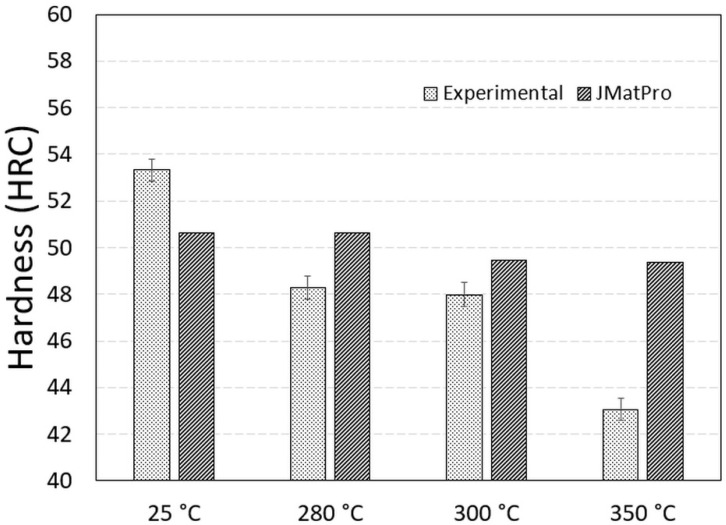
Average measured (experimental) and predicted (JMatPro) hardness (HRC) values according to heat treatment strategy from Figure 1.

The enhanced bainitic kinetics at higher temperatures are also shown in this paper for the given composition (Figure 4). The fraction of bainite depends on the chosen holding times and the position of the TTT diagram [19]. The importance of holding times during bainitization is also evident in [2] where hardness decreases with holding time due to decreased austenite content as a result of bainite formation and therefore decreased martensite fraction during cooling. Nevertheless, commercial JMatPro provides an opportunity to simulate and test the hardness relations of high-silicon grade steel with bainitic transformation for different cooling paths of laboratory tests and to compare the results to the measured ones. The thermodynamic prediction using JMatPro does not involve larger bainite fractions at 280 (0.049 vol.% of bainite), but they are significant at 300 °C (94 vol.% of bainite) and above, as it is above the fundamental Ms = 296 °C, as can be seen in Table 2. The JMatPro quench properties module was used to compare the measured hardness values of the heat-treated samples (according to the scheme in Figure 1). The overall trend of the hardness increasing with the isothermal temperature decreasing was observed using the measured and predicted hardness values. This was interpreted as being in good agreement with the predictions made by JMatPro by the increased fraction of martensite formed upon quenching at the given holding times. This is expected, as two experimental isothermal holdings were performed under the measured Ms. Additionally, the highest hardness values were obtained by direct quenching to develop a full microstructure of highly dislocated lath martensite, with 53 and 50 HRC for measured and predicted hardness, respectively, which can be seen in Figure 7. Each column of measured hardness in Figure 7 represents a mean of three measurements with the corresponding standard error using two rounds of trials for each heat treatment. The scatter is small, due to the very careful sample extraction. The location of samples extraction was evaluated before the heat treatment under the non-etched condition at the position 1/4T, as described in the Materials and Methods, to prevent scatter on behalf of the excessive casting defects usually coupled with increased segregations.

The difference between the prediction and experimental work is obvious at a holding temperature of 350 °C with 49 HRC (473 HB) and 43 (402 HB) in the pure bainitic region by considering the TTT diagram as shown in Figure 3b. This means that a temperature of 350 °C is not suitable for the target hardness of a minimum of 47 HRC (450 HB). The effect of dislocation density on the hardness difference between the predicted and measured value seemed to be less dramatic even with a rather high temperature of 350 °C when considering complete bainite transformation, *V*_*b*_ → 1. Higher estimated hardness performed by JMatPro does not include an increased amount of possibly stable retained, untransformed, and soft austenite, due to the incomplete bainitic transformation reaction, as described in Figure 4 related to the carbon saturation limit in austenite for a given composition. The limited bainitic transformation was also observed by microstructure analysis presented further in the paper as complete bainite transformation was not observed for all investigated samples. While the importance of thermodynamic modelling is undisputed, and it can be utilised to achieve the desired microstructures and material properties, it is very important to understand the limitations. This study is a good example of how the microstructural components can be partially predicted, but effects such as self-tempering during isothermal annealing are difficult to predict; additionally, components such as bainite that form under partial interstitial diffusion are hard to predict. Therefore, the importance of experimental data is demonstrated throughout the paper.

No considerable changes between 280 °C and 1 h of holding time and 2 h at 300 °C were recorded, both by the predicted and measured hardness values. This is interesting as both holdings had different starting martensite fractions which were achieved in the dual-phase starting microstructure, as shown in Figure 5. Based on the predictions and experimental work, the medium carbon content of the presented steel can achieve sufficient hardness in rather short-time holdings under or close to Ms. It is expected that hardness can additionally be increased by shortening the holding times by limiting the extent of bainite transformation, *V*_*b*_, and enhancing the martensite fraction. However, the possible reduction of other material characteristics such as low-temperature toughness should be considered due to the increased content of brittle martensite formation. Both isotherms under the measured Ms resulted in approx. 48 HRC or 484 HV (or 460 HB) compared to the maximum measured as-quenched hardness of 53 HRC or 545 HV (or 532 HB). This means that the potential tensile strength range for the given composition and holding at 280 and 300 °C was between 1566 and 1583 MPa [35], making bainitization promising for wear application. Zhao et al. conducted experiments on a low carbon steel, and concluded that austempering below Ms increases tensile strength and decreases the amount of retained austenite, compared to austempering above Ms [18]. Xia et al. obtained the highest hardness for a low carbon steel when conducting a quench and temper, and the austempering lowered the hardness by 10% [36]. However, Suh et al. austempered a spring steel with 0.55 wt% C and obtained a 10% higher hardness with austempering than with quenching and tempering, that also resulted in higher wear resistance [37,38]. Higher wear resistance is not always associated with increased hardness but also with increased fracture toughness, as the harder the brittle martensite cracks, the more ductile the softer bainite smears are during wear tests, thereby decreasing the weight loss during wear tests [39]. The highest fracture toughness can be obtained at temperatures below those of Ms [40].

### 3.4. LOM and SEM-EDS Analysis

**Figure 8:**
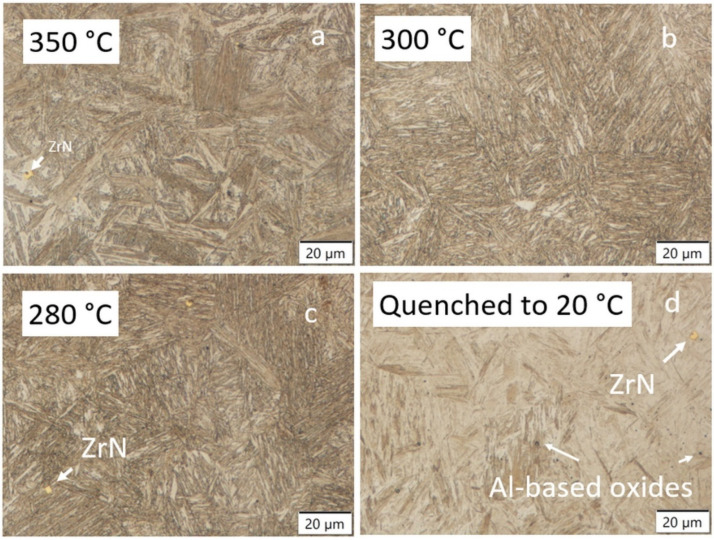
LOM of the dilatometric samples according to the heat treatment cycle presented in Figure 1. All samples austenitized at 920 °C with 20 min of holding time and isothermal holdings: (**a**) 350 °C, 2 h, (**b**) 300 °C, 2 h, (**c**) 280 °C, 1 h. (**d**) Quenched to 20 °C to obtain a fully martensitic structure.

The LOM examination of the microstructure for the isothermally treated samples between 350–280 °C can be challenging, as shown in Figure 8.

**Figure 9:**
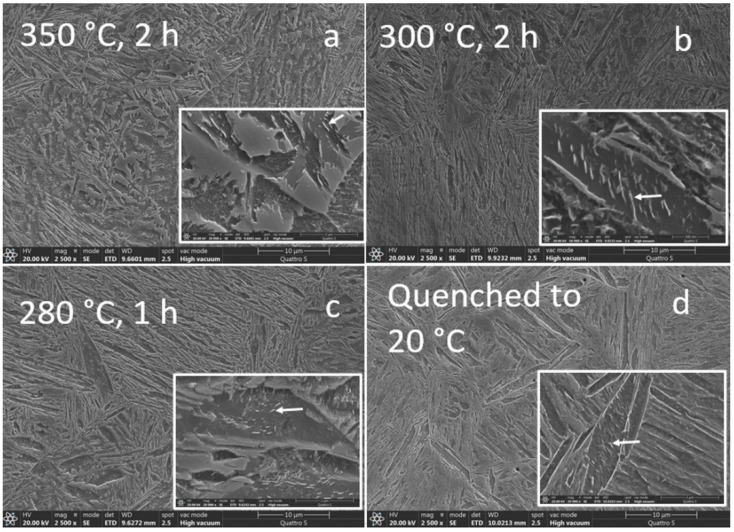
SEM of the dilatometric samples with marked carbides, heat treated according to the heat treatment cycle presented in Figure 1.

**Figure 10:**
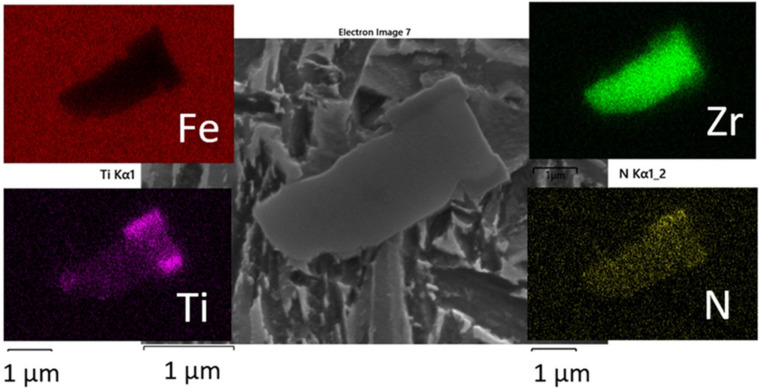
Coarse and complex particle, Zr (N, C) intermixed with Ti and therefore simplified written as Ti, Zr(N). The complex nitride has a cuboidal shape.

Nevertheless, the visual microstructure refinement with temperature decrease was made visible by comparing the microstructure formed after isotherm holding at 350 °C, 300 or 280 °C. The difference in the formed microstructure between 300 and 280 °C was barely noticeable using LOM and this agrees well with the similarity in the measured hardness. The etching effect using 5% Nital varied between the untempered hard athermal martensite (Figure 8d), and the softer mixed bainitic-martensitic structure formed during the isothermal holdings, as shown in Figure 8a–c. This mixed microstructure of martensite and bainite was also observed under Ms. Isothermal transformations under Ms were already observed to have a mixed type of bainitic-tempered martensitic structure [18]. The visual change in morphology using LOM between the fully quenched martensite and mixed bainitic-martensitic microstructure close to the Ms structure can easily be blurred with the additional low-temperature tempering of athermal martensite. Therefore, SEM was proposed for a more detailed microstructural investigation to distinguish between the possible carbide-free upper and lower bainite and also to observe the bainite sheaves, self-tempered martensite, and evidence of the possible blocky morphology and distribution of the coarse austenite-martensite formed between the sheaves of bainite. It is also possible to observe the presence of coarse (Zr, Ti)N non-metallic inclusions formed during solidification using LOM. Zr-based particles (simplified written as ZrN) were recognized due to the typical yellow color after Nital etching, as presented in Figure 8. So, they do not act as pining particles, as demonstrated by the relatively coarse final microstructure. Zirconium addition in low carbon steels reduces the level of oxygen, nitrogen, sulphur, and potentially carbon in the matrix. Fine oxides dispersed in steel and also nitrides and carbo-nitrides can have a strong influence on the final characteristics of the product in terms of the processing technology used [41]. When the oxygen is consumed, nitrides start to form and, if precipitation is above T_sol_, zirconium-based non-metallic inclusions are formed [42], which are observed as coarse particles in a martensitic and/or bainitic matrix, visible using LOM (as a yellow cubic particle) and more clearly observed by SEM, as presented in Figure 8, Figure 9 and Figure 10. Using the solubility data taken from [41,43], the precipitation of ZrN begins above T_sol_. It is believed that the small oxides can influence the nucleation of acicular ferrite in the heat-treated condition and also in the as-cast state, but they do not pin the grain boundaries [41]. This means that even by introducing micro-alloying, the final PAGS according to the results is still coarse regardless of the chosen heat treatment step.

Hardness contribution is associated with the formed particles, as a part of the primary and secondary precipitation, achieved dislocation density of phase constituents concerning grain size, and in the case of martensitic microstructure, prior austenite grain size, and the presence of various phase constituents and related volume proportions. The latter seems to be most important, as described by the linear Equation (4). In Figure 8a and Figure 9a (350 °C), a mixed martensitic to bainitic microstructure is observed. From the morphological view, this type of microstructure is considered as heterogeneous. By decreasing the holding temperatures, a microstructure, which visually seems more homogeneous but is still composed of martensite and bainite, is formed, and according to Figure 8b, c the microstructure formation is closer to a self-tempered state. An increased tempering effect is obvious at 280 and 300 °C compared to quenched martensite formation (Figure 9d) with respect to the density of carbides and related etching effects. In the case of 350 °C, the precipitation of carbides or the carbide density is less pronounced compared to the lower holding temperatures at 300 and 280 °C. This could be related to the lack of martensite formation above Ms and the influence of high silicon addition which acts as a carbide inhibitor. However, the presence of carbides is probable despite the silicon content in all samples. This is in agreement with observations where tempered martensite and lower bainite both contained cementite particles even with approx. 2 wt% Si [7] or in the case of Wang [28] ɛ-carbides were found in 0.24 wt% C and 1.8 wt% Si steel tempered at 250 °C. It was recognized that a small number of needle-like carbides were probable also in the quenched state with a similar carbon content to the decreased silicon content [1].

At 350 °C, the martensitic-austenitic (MA) blocky islands were coarse, suggesting that sufficient unstable residual austenite was present before final quenching, due to the unfinished bainitic transformation, regardless of the faster bainitic kinetics at higher temperatures. More detailed investigations using SEM revealed that low density (nano) precipitates exist in practically all investigated samples, regardless of the high silicon content (2.0 wt%) and that the particles are less than 100 nm in thickness and under 500 nm in length. At the highest holding temperatures, carbides are no longer needle-like, but more spherical in geometry and are already hard to observe as already emphasized. The presence of the high-density needle-like carbides in the similar as-quenched form and high silicon steel with 0.3 wt% C and 1.6 wt% Si and 3.5 wt% Mn was also noticed in the coarse-grained structure, where no clear carbide formation was noticed in the highly refined structure using a 50 K/s quenching rate [44], thereby emphasizing the importance of grain size. In the case of 0.30 wt% C, 1.57 wt% Mn carbide precipitation was suppressed at holding at 350 °C from 10 to 150 min [19].

In the last solidification front (inter-dendritic regions), coarse hard nitrides were observed as non-metallic inclusions, of more than 1 µm in size. An example of the coarse and complex primary nitride or carbonitride with titanium co-precipitation is presented in Figure 10. The size was taken to be ineffective for grain size control, as discussed before. The study of minor additions of zirconium in titanium-based or titanium-niobium based HSLA confirmed the presence of complex Ti-Zr nitrides, rather than ZrN, typically of more than 1 µm in size [29] or up to 10 µm in the vanadium-zirconium microalloying system [31], where zirconium-based carbonitrides also had titanium traces. In this study, the titanium was not intentionally added and was assumed to originate from the Zr-based wire (with minor titanium addition). The size of the Zr-based nitrides (carbonitrides) was limited to up to 5 µm.

## 4. Conclusions

By introducing the isothermal quenching of the medium carbon and high silicon steel casting, the required minimum 450 HB (47 HRC) was achieved on samples treated only at very near to Ms (321 °C), with maximum hardness achieved by direct quenching forming partially self-tempered lath martensite (53.3 HRC). The calculated hardness using commercial software indicated that all routes could achieve the required hardness, as was also the case for holdings in the bainite region of much above Ms. The predicted value for direct quenching was too low at 50.6 HRC compared to the experimental 53.3 HRC. Meanwhile the predicted values for bainitization were too high at 50.6 HRC, with an experimental value of 48.3 HRC for 280 °C, 49.4 HRC and an experimental value of 48 HRC for 300 °C, and finally 49.4 HRC and an experimental value of 43 HRC for 350 °C. This discrepancy between predictions and experimental work appeared mainly at the highest holding temperature of 350 °C and seemed to originate from the unfinished bainite transformation (with low fraction of fine sized *V*_*b*_) and subsequent presence of sufficient soft residual austenite (*V*_γ_) among partially transformed austenite into martensite found at room temperature. A lower measured hardness after cooling from the highest holding temperature at 350 °C (43 HRC), compared to other holdings, was also partially related to the lack of a higher population density of carbides and potentially lower dislocation density achieved in the final microstructure. Holding the samples at 280 °C and 300 °C resulted in 48.3 and 48 HRC.

The sensibility of the internal thermodynamic and physical data in the used commercial program regarding hardness contributions of bainite itself is rather minor as it is known that increasing the upper bainite fraction decreases hardness substantially compared to highly dislocated martensite at a low alloyed steel grade. The coarsest microstructure was obtained by performing bainitization at 350 °C, with evidence of coarse bainite and coarse martensitic-austenitic (MA) blocky islands and was the product of the residual and unstable austenite present before final quenching. High-magnification SEM investigation also revealed the presence of intra low-density carbides that accompany lower bainite transformation with coarse PAG size, as is common for castings. They are recognized as needle-shaped carbides and are therefore not related to the non-sufficient carbide solubility achieved at the given austenitization temperature and holding time before quenching. According to the laboratory sets, the most refined structure was observed when holding the sample close to or under Ms. Due to the relatively coarse PAG of as-cast steel, no difference in the change of bainitic kinetic behavior was observed by changing the holding time at austenitization temperature.

Thermodynamic modelling is a very good tool to determine Ms and plan bainitization isothermal holding temperatures, but the available software does not allow for the accurate prediction of mechanical properties for bainitic microstructures.

## Figures and Tables

**Table 1:** Chemical composition of investigated as-cast steel grade (wt%).

C	Si	P	S	Mn	Cr + Mo	Cu + Ni	Fe
0.32	2.0	0.015	0.006	1.58	0.99	0.16	rest

**Table 2:** Measured and predicted characteristic temperatures of AR casting.

	Ac1_/_e1	Ac3_/_e3	Ms	Mf	Bs
Experimental	760	830 *	321	180	-
Thermo-Calc	722	820	324 **	200 ***	-
JMatPro	731	826	296	169 ****	486
Mucg83	-	-	324	-	488

* Highest measured temperature to incorporate the influence of the non-homogenized as-cast state. ** With corrected parent phase Gibbs energy addition. *** For 99 vol. % of martensite formation calculated by Thermo-Calc. **** For 90 vol. % of martensite formation calculated by JMatPro without corrections.
